# CD81 and Its Relationship to Treatment Response in Patients With Acute Myeloid Leukemia at a Hospital in Hanoi, Vietnam

**DOI:** 10.7759/cureus.40245

**Published:** 2023-06-11

**Authors:** Tung Tuan Nguyen, Le Lan Anh, Quang Ha Hong, An Thi Vinh Do

**Affiliations:** 1 Hematology and Blood Transfusion, Bach Mai Hospital, Hanoi, VNM

**Keywords:** hematology, bone marrow, prognosis, acute myeloid leukemia, cd81

## Abstract

Background: Acute myeloid leukemia (AML) has the proliferation of poorly differentiated immature myeloid cells. New studies on immune markers also consider them as one of the factors that affect the prognosis or the patient's ability to respond to drugs. Our study was designed to determine the rate of remission and mortality, and the ability to respond to drugs in newly diagnosed AML patients with positive CD81.

Methods: A total of 50 patients diagnosed with AML (excluding acute promyelocytic leukemia) underwent immunophenotyping analysis using flow cytometry. Following the initial diagnosis, the patients received induction therapy, followed by three cycles of consolidation therapy. The patients were then followed up for a period of six months. The treatment efficacy was assessed at two timepoints: on day 28 after the first chemotherapy course and on day 28 after the fourth chemotherapy course.

Results: Out of the 50 newly diagnosed AML patients, 40 (80%) were found to be CD81 positive. This CD81-positive group had a high mortality rate after the first course of chemotherapy (17.5%) and after the fourth course of chemotherapy (52.5%), while no patients died in the CD81-negative group. The CD81-positive group had a worse drug response rate with 22.5% and 18.2% in CD81 positive group versus 30% and 40% in the CD81-negative group achieving complete remission after the first course and fourth course, respectively.

Conclusions: The CD81 immunological marker was found to be highly prevalent among AML patients in Vietnam. Overexpression of CD81 in patients with AML is associated with an unfavorable prognosis, characterized by higher mortality rates and poorer treatment response.

## Introduction

Overview

Acute myeloid leukemia (AML) is characterized by the uncontrolled proliferation of myeloid blast cells, resulting in the failure of normal hematopoiesis in the bone marrow [1]. Currently, the 3+7 combination regimen (three days of daunorubicin and seven days of cytarabine) is still accepted as the cornerstone of initial treatment for AML patients in 70-80% of cases [2]. However, recurrence still occurs in about half of patients diagnosed with AML, and the five-year survival rate is only about 40% [3]. Over the past few years, identifying new prognostic markers has remained important; especially in influencing the decision to choose treatment [4]. Several chromosomal and gene abnormalities (eg, *NPM1*, *CEBPA*, and *FLT3-ITD* mutations) have been used in clinical practice [4]. However, clinicians find that many patients with good chromosomal and genetic prognosis still have a poor response to treatment or relapse rapidly, which asks the question of whether there are other factors that determines the prognosis of AML patients.

CD81 is a cell surface protein of the tetraspanin family, which is a cell surface transmembrane protein. The CD81 antigen belongs to the tetraspanin family, which are cell surface transmembrane proteins. This antigen was initially detected as the target of an anti-proliferative antibody and was later named TAPA-1 [5]. It binds to other proteins in dynamic membrane entities known as tetraspanin-enriched microdomains (TEMs) and to receptors that vary by cell line (eg, CD19 in B lymphocytes) [6]. Various cellular functions are linked to CD81 (i.e. BCR signaling in B cells [7], interacting B-T cells, and cell entry receptor function for infectious diseases [8,9]. Therefore, TAPA-1 may play an important role in the regulation of cell growth in lymphoma patients [5]. Vences-Catalan and colleagues demonstrated the dominant role of CD81 affecting metastasis and immunoregulatory in cancer [10].

Flow cytometry has been applied in Vietnam since the 2000s. However, the appearance of CD81 on the surface of myeloid blast cells has not been studied in Vietnam. Therefore, this study was designed with the aim of determining the prognostic significance of CD81 in patients with AML.

## Materials and methods

This study included 50 newly diagnosed AML patients (except acute promyelocytic leukemia) who had an examination and were given treatment at Bach Mai Hospital, Hanoi, Vietnam, from September 2021 to December 2022. Informed consent was obtained from all participants.

Methods

We used a convenient sampling method in order to take all patients with newly diagnosed AML (except acute promyelocytic leukemia) during the study period. A list of investigations was performed, including clinical examination, complete blood count (CBC), bone marrow aspiration, chromosomal karyotype analysis, and immunophenotyping of the bone marrow sample by flow cytometry (FCM) using Navios EX Flow Cytometer (Beckman Coulter Inc., Brea, California, United States) with the following markers: MPO, CD13, CD33, HLA-DR, TdT, CD4, CD64, CD34, CD3, CD19, CD20, CD22, MPO, CD2, and CD81.

The patients were treated with induction therapy of a 3+7 regimen consisting of three days of doxorubicin infusion (30 mg/m^2^) in combination with seven consecutive days of cytarabine infusion (100 mg/m^2^) daily continuously, after that the patients continued to receive consolidation therapy with high dose cytarabine (HiDAC) for three courses.

Bone marrow aspiration was performed on day 28 after receiving the first and after the fourth chemotherapy treatment to evaluate the rate of complete remission (CR), no complete remission (no CR) and mortality, and the ability to respond to drugs

Data processing

The data were managed and analyzed using IBM SPSS Statistics for Windows, Version 20.0 (Released 201; IBM Corp., Armonk, New York, United States). Descriptive statistics: number (n%); inferential statistics: p<0.05 has statistical significance.

Research ethics

The Ethics Committee of Bach Mai Hospital approved the study protocol and documents related to the research procedures (approval number: 3666-/BM- HDDĐ). All patient information was kept strictly confidential and used for research purposes only. The CD81 marker was performed in the Flow cytometric acute leukemia panel and, therefore, there was almost no risk to the health of the study participants or any financial cost. This is a purely observational study, so it does not affect the patient's treatment process.

## Results

Our results included 40/50 (80%) CD81-positive AML patients with an average age of 56.08±15.09, which was lower than CD81-negative patients with an average age of 66.60±11.37 ( p<0.05). Besides, the M2 and M4 forms had the highest rate (Table 1).

**Table 1:** Clinical characteristics of study subjects FAB: French-American-British classification

Clinical characteristics	CD81-positive (n=40)	CD81-negative (n=10)	p-value
Age	56.08±15.09	66.60±11.37	0.045
Gender			0.289
Male	18 (45.0%)	7 (70%)	
Female	22 (55.0%)	3 (30%)	
FAB subtype			0.239
M0	4 (10%)	2 (20%)	
M1	6 (12.5%)	1 (10%)	
M2	11 (27.5%)	2 (20%)	
M4	13 (32.5%)	2 (20%)	
M5	7 (17.5%)	0	
M7	0	1 (10%)	
Anemia	40 (100%)	10 (100%)	-
Bleeding	22 (55.0%)	2 (20.0%)	0.001
infection	29 (72.5%)	4 (40.0%)	0.07

Anemia was the most common symptom in acute leukemia, with 100% of patients presenting with anemia. In the CD81-positive AML group, the mean hemoglobin (Hb) concentration was 70.38±17.82 (g/l) and the mean platelet count was 46.81±37.64 (G/L) (Table 2).

**Table 2:** CBC characteristics of study subjects CBC: complete blood count

CBC	CD81-positive (n=40)	CD81-negative (n=10)	p-value
Hemoglobin (g/l)	70.38±17.82	83.14±15.61	0.091
Platelet count (G/l)	46.81±37.64	106.14±81.77	0.008
White blood count (G/l)	86.04±115.00	18.02±11.49	0.133
Percentage of blast cells (%)	38.42±30.62	28.86±26.28	0.428

When studying cytogenetics, we found that many patients had several chromosomal formula abnormalities including t(8;21), inv(16), and others (Table 3).

**Table 3:** Chromosomal karyotype analysis of the bone marrow p=0.663 (Fisher's exact test)

	Normal	t(8;21)	Inv(16)	Aneuploid	Polyploidy	Others	Total
CD81+	31 (77.5%)	1 (2.5%)	2 (5.0%)	0	2 (5.0%)	4 (10.0%)	40
CD81-	9 (90.0%)	0	0	1 (10.0%)	0	0	10

Patients who did not recover after the first course of chemotherapy were 31/50 and those who did not recover after the fourth course were 19/40; this is a high rate with a poor prognosis. Moreover, there were 21 patients died after the fourth course, all of them were CD81 positive (Table 4-5). 

**Table 4:** Results of treatment after being given the first therapy course p=0.409 (Fisher's exact test) CR: complete recovery

	CR	No CR	Dead
CD81+ (n=40)	9 (22.5%)	24 (60.0%)	7 (17.5%)
CD81- (n=10)	3 (30.0%)	7 (70.0%)	0

**Table 5:** Results of treatment after being given the fourth therapy course Fisher's exact test

	CR	No CR	Dead	p-value
CD81+ (n=40)	6 (15.0%)	13 (32.5%)	21 (52.5%)	0.004
CD81- (n=10)	4 (40.0%)	6 (60.0%)	0	

## Discussion

Our study group includes 50 patients, including 40 CD81-positive patients, with an average age of 56.08±15.09, lower than CD81-negative patients with an average age of 66.60±11.37 (p<0.05).

Anemia is the most common symptom in acute leukemia, with 100% of patients presenting with anemia, in the CD81-positive AML group, the mean Hb concentration was 70.38±17.82, which was lower than that in the CD81-negative AML group. The patients in the study, whether CD81 positive or negative, had other clinical complications such as infection and bleeding, and the rates were not too different among the two groups. When studying cytogenetics, we found that the patients had a high percentage of normal chromosomal karyotype, other abnormalities were found and were similar to the study on chromosomal abnormalities in acute leukemia patients by Quang in Vietnam in 2003 [11].

In our study, up to 40/50 newly diagnosed AML patients were CD81-positive, accounting for 80%; this result was higher than the study results of Boyer et al. (69%) and Hussein et al. (17/30 patients) [12,13]. The CD81-positive group had a high mortality rate on day 28 after being given the first chemotherapy course, up to 7/40 (17.5%) patients, while no patients died in the CD81-negative group. At the end of the fourth course, the mortality rate in the CD81-positive group was still very high (52.5%), while in the CD81-negative group, no patients died. Regarding drug response, with a minimum residual rate of <5.0% blast cells, the CD81-positive group also had a worse drug response rate, with 9/40 (22.5%) and 6/40 (15.0%) achieving CR after the first and the fourth chemotherapy courses, respectively. Besides, in the CD81-negative group, the percentage of patients achieving CR after the first and the fourth treatment courses were 3/10 (30%) and 4/10 (40%), respectively.

In previous studies, CD81 was found to be present in the plasma cells of patients diagnosed with multiple myeloma, resulting in faster relapses, and a worse prognosis for progression-free survival (PFS) and overall survival (OS) [14]. Not only that, but CD81 can also be a poor prognostic marker in AML [15]. In 2016, Boyer et al. studied the appearance of the CD81 marker on the surface of myeloid leukemia cells related to the survival prognosis of patients [12]. A recent study by Hussein et al. also showed that the presence of CD81 led to low survival time and fast recurrence in AML patients [13]. Gonzales et al., in an in vivo and in vitro study, found a 30-40% rate of resistance to daunorubicin and cytarabine in CD81-positive AML patients [16]. In our study, CD81-positive AML patients had a higher mortality rate (52.5%) than the CD81-negative group (0%). Among the CD81-positive AML patients who died in our study, there was a mutation in the *NPM1*(+) gene, which is a good prognostic mutation, but this patient did not immediately recover from the first course of chemotherapy. This may be because CD81 is a cell surface protein of the tetraspanin family, which is a cell surface transmembrane protein. In a clinical trial of the monoclonal antibody tetraspanin in mice, it was found that the monoclonal antibody was able to induce an antiproliferative effect on the human lymphoma cell line [5], leading to the uncontrolled proliferation of malignant cells. Previously, in Paiva's study, it was found that in multiple myeloma, the detection of CD81 positivity on plasma cells was a poor prognostic factor [14]. In Hussein et al.'s study in 2021, eight CD81-positive patients (26.6%) died during chemotherapy treatment courses, while only two CD81-negative patients (6.7%) died. These results could be explained by the finding of Boyer et al., who demonstrated that CD81-positive malignant cells were 30-50% more resistant to drugs and that overexpression of CD81 increased AML cell adhesion, which induced blast number and caused relapses [17]. Regarding the response to chemotherapy, Hussein et al. found that 50% (15/30) of the patients with CD81 positive expression achieved CR, while 43% did not respond to treatment. This result was also similar to our assessment, that patients with CD81 expression had a statistically shorter remission and shorter survival time than CD81-negative patients [16]. 

Unfortunately, in this study, we encountered some limitations. Some patients did not do enough tests to find out the presence of prognostic genetic mutations because they could not afford it. 

## Conclusions

In the year-long study with 50 AML patients, up to 80% of the patients were CD81-positive. The group of CD81-positive patients had a shorter survival time and a higher rate of blast disease recurrence than the CD81-negative group, even in patients with good prognostic genes. As can be seen, CD81 overexpression in patients with AML is associated with a poor prognosis, higher mortality, and poorer response. The addition of CD81 in the AML panel can be considered as an essential CD required for prognostic assessment.

## References

[1] Papaemmanuil E, Gerstung M, Bullinger L (2016). Genomic classification and prognosis in acute myeloid leukemia. N Engl J Med.

[2] Döhner H, Weisdorf DJ, Bloomfield CD (2015). Acute myeloid leukemia. N Engl J Med.

[3] Deschler B, Lübbert M (2006). Acute myeloid leukemia: epidemiology and etiology. Cancer.

[4] Döhner H, Estey EH, Amadori S (2010). Diagnosis and management of acute myeloid leukemia in adults: recommendations from an international expert panel, on behalf of the European LeukemiaNet. Blood.

[5] Oren R, Takahashi S, Doss C, Levy R, Levy S (1990). TAPA-1, the target of an antiproliferative antibody, defines a new family of transmembrane proteins. Mol Cell Biol.

[6] Levy S (2014). Function of the tetraspanin molecule CD81 in B and T cells. Immunol Res.

[7] Mattila PK, Feest C, Depoil D (2013). The actin and tetraspanin networks organize receptor nanoclusters to regulate B cell receptor-mediated signaling. Immunity.

[8] Mittelbrunn M, Yáñez-Mó M, Sancho D, Ursa A, Sánchez-Madrid F (2002). Cutting edge: dynamic redistribution of tetraspanin CD81 at the central zone of the immune synapse in both T lymphocytes and APC. J Immunol.

[9] Charrin S, le Naour F, Silvie O, Milhiet PE, Boucheix C, Rubinstein E (2009). Lateral organization of membrane proteins: tetraspanins spin their web. Biochem J.

[10] Vences-Catalán F, Rajapaksa R, Srivastava MK, Marabelle A, Kuo CC, Levy R, Levy S (2015). Tetraspanin CD81 promotes tumor growth and metastasis by modulating the functions of T regulatory and myeloid-derived suppressor cells. Cancer Res.

[11] Quang VP (2003). Study of Chromosomal Abnormalities in Acute Leukemias in Adults at the Institute of Hematology (Thesis in Vietnamese). Hanoi Medical University, Vietnam.

[12] Boyer T, Guihard S, Roumier C (2016). Tetraspanin CD81 is an adverse prognostic marker in acute myeloid leukemia. Oncotarget.

[13] Hussein O, Ahmed R, Fathy A, Salah H (2021). Prognostic role of tetraspanin CD81 in patients with acute myeloid leukemia. ZUMJ.

[14] Paiva B, Gutiérrez NC, Chen X (2012). Clinical significance of CD81 expression by clonal plasma cells in high-risk smoldering and symptomatic multiple myeloma patients. Leukemia.

[15] Khwaja A, Bjorkholm M, Gale RE (2016). Acute myeloid leukaemia. Nat Rev Dis Primers.

[16] Gonzales F, Boyer T, Peyrouze P (2017). Targeting aberrant expression of CD81 impacts cell adhesion and migration, drug resistance and prognosis of acute myeloid leukemia. Blood.

[17] Boyer T, Gonzales F, Plesa A (2018). Flow cytometry to estimate leukemia stem cells in primary acute myeloid leukemia and in patient-derived-xenografts, at diagnosis and follow up. J Vis Exp.

